# OUTCOMES OF ASSISTED LIVING ACCREDITATION IN NORTH CAROLINA

**DOI:** 10.1093/geroni/igae098.0505

**Published:** 2024-12-31

**Authors:** Sheryl Zimmerman, Lea Efird-Green, Philip Sloane, David Reed, Yuke Tian, Scott Davis, John Preisser

**Affiliations:** University of North Carolina at Chapel Hill (UNC), Chapel Hill, North Carolina, United States; University of North Carolina at Chapel Hill, Chapel Hill, North Carolina, United States; University of North Carolina, Chapel Hill, North Carolina, United States; University of North Carolina, Chapel Hill, North Carolina, United States; University of North Carolina, Chapel Hill, North Carolina, United States; University of North Carolina Eshelman School of Pharmacy, Asheville, North Carolina, United States; University of North Carolina, Chapel Hill, North Carolina, United States

## Abstract

Accreditation is a widely used health care review process to determine if an organization meets a defined standard of quality. The opportunity for accreditation recently moved into the field of assisted living (AL); today, seven states have statutes and regulations allowing third party accreditation to satisfy full or partial compliance with state licensure or certification. Data indicate that compliance with accreditation standards may result in improved care, but it has not been evaluated in AL. In 2021, the state of North Carolina funded an AL accreditation pilot program to evaluate whether accreditation improves or maintains quality. Communities were randomly allocated to a control or accreditation arm and are being followed for two years to evaluate care and outcomes in five areas (workforce, resident outcomes, care coordination and transitions, medication management, person-centered care). Of the 146 communities originally enrolled that provided data, most are for-profit (96%) and part of a chain (73%); 44% pf residents have dementia, 25% have mental illness, and 53% receive state financial assistance. Preliminary results in the first two quarters (Q) found an overall increase in advance care planning discussions (53% in Q1 and 63% in Q2), and a decrease in medication administrations with one or more errors (1.2% in Q1 and 0.6% in Q2). This session will present results comparing communities in the control arm, accreditation arm, and those that successfully achieved accreditation, with findings having wide-ranging implications for the future of AL accreditation and regulation in North Carolina, and implications across the country.

